# Is the Even Distribution of Insecticide-Treated Cattle Essential for Tsetse Control? Modelling the Impact of Baits in Heterogeneous Environments

**DOI:** 10.1371/journal.pntd.0001360

**Published:** 2011-10-18

**Authors:** Steve J. Torr, Glyn A. Vale

**Affiliations:** 1 Natural Resources Institute, University of Greenwich, London, United Kingdom; 2 South African Centre for Epidemiological Modelling and Analysis (SACEMA), University of Stellenbosch, Stellenbosch, South Africa; Johns Hopkins Bloomberg School of Public Health, United States of America

## Abstract

**Background:**

Eliminating Rhodesian sleeping sickness, the zoonotic form of Human African Trypanosomiasis, can be achieved only through interventions against the vectors, species of tsetse (*Glossina*). The use of insecticide-treated cattle is the most cost-effective method of controlling tsetse but its impact might be compromised by the patchy distribution of livestock. A deterministic simulation model was used to analyse the effects of spatial heterogeneities in habitat and baits (insecticide-treated cattle and targets) on the distribution and abundance of tsetse.

**Methodology/Principal Findings:**

The simulated area comprised an operational block extending 32 km from an area of good habitat from which tsetse might invade. Within the operational block, habitat comprised good areas mixed with poor ones where survival probabilities and population densities were lower. In good habitat, the natural daily mortalities of adults averaged 6.14% for males and 3.07% for females; the population grew 8.4× in a year following a 90% reduction in densities of adults and pupae, but expired when the population density of males was reduced to <0.1/km^2^; daily movement of adults averaged 249 m for males and 367 m for females. Baits were placed throughout the operational area, or patchily to simulate uneven distributions of cattle and targets. Gaps of 2–3 km between baits were inconsequential provided the average imposed mortality per km^2^ across the entire operational area was maintained. Leaving gaps 5–7 km wide inside an area where baits killed 10% per day delayed effective control by 4–11 years. Corrective measures that put a few baits within the gaps were more effective than deploying extra baits on the edges.

**Conclusions/Significance:**

The uneven distribution of cattle within settled areas is unlikely to compromise the impact of insecticide-treated cattle on tsetse. However, where areas of >3 km wide are cattle-free then insecticide-treated targets should be deployed to compensate for the lack of cattle.

## Author Summary

Eliminating Rhodesian sleeping sickness, the zoonotic form of Human African Trypanosomiasis found in East and Southern Africa, can be achieved only through eliminating the vectors, species of tsetse fly (*Glossina*). The deployment of insecticide-treated cattle is the most cost-effective means of achieving this. However, the even distribution of insecticide-treated cattle is seldom possible due to the patchy distribution of grazing, water and human settlement. We used a simulation model to explore the likely impact of such patchiness on the outcome of control operations against tsetse. The results suggest that even in areas that are highly suitable for tsetse, gaps of up to 3 km in the distribution of insecticide-treated cattle will not have a material impact on the success of an operation provided the overall mean density of cattle across all areas is adequate to achieve control (e.g., ∼4 insecticide-treated cattle/km^2^ killing 10% per day of the tsetse in the area treated). If the gaps are larger than 3 km, then deploying insecticide-treated targets at densities of 4/km^2^ in the cattle-free areas will ensure success.

## Introduction

Rhodesian sleeping sickness, caused by *Trypanosoma brucei rhodesiense*, is transmitted by tsetse flies (*Glossina* spp.) across East and Southern Africa. The disease is the zoonotic form of Human African Trypanosomiasis (HAT) in which the trypanosomes are harboured by reservoir hosts, primarily in wild and domestic suids and bovids. As a consequence, treating humans only cannot eliminate the disease. Rather, in addition to treating people carrying HAT, interventions must also be directed at removing trypanosomes from reservoir hosts and eliminating the vectors [1]. In cases where the reservoirs are wild mammals, the only practicable option is to control tsetse. Tsetse also transmit species of trypanosome (*T. vivax*, *T. congolense*, *T. simiae* and *T. b. brucei*) that cause Animal African Trypanosomiasis in livestock. Every year, more than a million cattle are killed by tsetse-transmitted trypanosomiases across sub-Saharan Africa, despite $30–40 million being spent annually on veterinary trypanocides [2]. The development and application of more cost-effective methods of tsetse control [3]–[5] combined with a strengthened political resolve across sub-Saharan Africa to tackle trypanosomiasis [6] has revived interests in interventions against tsetse [7], [8].

Insecticidal techniques for controlling tsetse flies (*Glossina* spp.) have been used successfully for 60 years, in many tens of thousands of square kilometres [9]. However, the control has not always proceeded as quickly and efficiently as required, for two main reasons. First, invasion from untreated areas nearby can re-infest all or much of the cleared territory [10], as after aerial spraying in Botswana during the 1980s [11]; this problem can be solved by creating invasion barriers of odour-baited targets treated with insecticide [4], [12], [13]. Second, the control measures have not always been applied at the same time and intensity throughout the operational area, so that residual pockets of infestation remain, as in the early aerial spraying operations in Botswana [11] and some of the ground spraying in Zimbabwe [14]. The difficulty of even cover can be particularly serious when control is based on pyrethroid-treated cattle since the animals available for treatment are often distributed patchily, due to the animals' need for adequate grazing and water [15], [16]. This is unfortunate since the cattle treatment is by far the most economical method of control [3], [17].

Finding solutions to the above problems should, ideally, refer directly to abundant data from a full range of technical options tried previously in a wide variety of circumstances. However, such data are scant since full population monitoring is a luxury achievable mainly during the first few trials with a new technique [14]. Moreover, to identify confidently the limits to a technology it is necessary to use it above and below the limits. Understandably, practitioners do not deliberately attempt something that might fail. If failure does occur, by mere happenstance, actions are taken to correct the problem quickly by any means available, as when dealing with pockets of infestation left by aerial spraying in Zimbabwe [14], Botswana [4] and Somalia (S. Torr, unpublished data). Thus, there are few opportunities to assess accurately the number, distribution and dynamics of flies in the problem situations, especially since the populations there are typically sparse and hence difficult to sample.

To offset the paucity of data from actual field campaigns, we have much basic information for population dynamics in the field and laboratory [18], so allowing the modelling of tsetse control [10], [19], [20]. Previously we have used the simulation programme ‘Tsetse Muse’ [20] to assess the relative cost-effectiveness of insecticide-treated cattle and the sterile insect technique [20], and the performance of aerial spraying in Botswana [4]. The present paper employs Tsetse Muse to assess how the heterogeneous distribution of baits (insecticide-treated cattle and targets) affects their impact on tsetse populations, and how residual pockets of infestation might be avoided and/or eliminated.

## Materials and Methods

### Model

**Figure 1:**
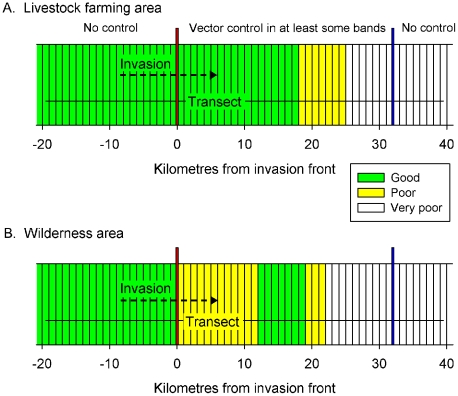
Modelled transect though livestock farming or wilderness areas where tsetse control operations were conducted. The mapped areas consist of imaginary bands, 1 km wide, extending for many kilometres up and down the page. Other bands were present to an effectively infinite distance to the left and right, but they are not shown. For both livestock farming (A) and wilderness (B) scenarios, bands to the left of the invasion front (red vertical line, 0 km) were good habitat while those to the right comprised mixtures of good (green bands), poor (yellow bands) and very poor (white bands); at 32 km from the invasion front the density of males declined to <0.1/km^2^ (back edge, vertical blue line). All operations were applied between the invasion front and the back edge and hence subject to invasion from a tsetse population in good habitat to the left of the invasion front.

The model is detailed by [20] and can be downloaded at www.tsetse.org and the parameters adopted for its present use are indicated in Table S1. It will be only summarised here. The numerical and spatial distributions of population components were tracked deterministically using the spreadsheet programme Microsoft Excel 2003, it being taken that the population occurred in parallel bands of habitat that were 1 km wide, with the habitat being uniform within bands but allowed to differ between bands (Fig. 1). Outputs showed the abundance of insects along a transect that ran straight across the bands.

#### Standard population

This population, occupying extensive blocks of good (i.e., highly suitable for tsetse) habitat, consisted of 2500 adult males/km^2^ and 5000 adult females/km^2^. Daily adult mortalities were age-dependent, averaging 6.14% for males and 3.07% for females, with maximum adult life spans of 89 and 178 days, respectively. In this paper *mortality* is taken as the complement of the *survival probability* and expressed, for ease of understanding, as a percentage. Mortality during the egg and larval period was set at 5%. Males were sexually mature at five days and females at three. The first larva was produced at age 16 days and the interlarval period was 9 days. The pupal duration was 28 days for males and 26 days for females, with a mortality of 25% during this period. Males and females emerged in equal numbers. Daily displacement of adults averaged 249 m for males and 367 m for females, to suit the field data for many tsetse species [21].

#### Density dependence

Natural mortality of adults, pupae and eggs/larvae in each band declined linearly with reduction in population density, to be steady at 75% of standard values when the population density was ≤10% of standard – the density reference for the mortality of adults and eggs/larvae being the abundance of all adults, and for pupae being pupal abundance. Females found mates with a daily probability of 0.1 if there was only one mature male/km^2^. Provided the abundance of males was not critical, an isolated population could increase up to 8.4 times per year, *i.e.*, roughly the rates observed for island populations [18]. If the male density was <0.1/km^2^ the population expired naturally.

#### Control methods

All simulated control was performed by baits that killed a constant percent of adults per day, albeit that the percent was varied between simulations. The percent was often set at 10%, to represent the percentages achievable against *Glossina morsitans morsitans* Westwood and *G. pallidipes* Austen by the use of artificial baits, *i.e.*, traps or insecticide-treated targets deployed at about 5–10/km^2^ [14], [20], or by the application of insecticide to cattle [3], [16]. The value was sometimes allowed to be 50% this being the likely maximum achievable given that tsetse feed at minimum intervals of about two days. This theoretical maximum could be somewhat lower since some tsetse might feed on hosts other than cattle, and some tsetse contacting the treated animals might not die [3]. In approximate compensation for this, tsetse visit cattle several times during the hunger cycle [22], thereby increasing the chance of being killed. Unless stated otherwise, all control in a band ceased when the density of males there declined to <0.1/km^2^, *i.e.*, the point at which the population was not self-sustaining.

Although sparse populations were not self-sustaining, they did not expire if supplemented by invasion. Moreover, such populations were in principle detectable, especially since the modelling showed that there were often many times more females than males. Hence, it was assumed that the population would be undetectable in practice only when the density of adult females dropped to 0.1/km^2^. At this density, if survey traps were operated with an individual probability of catching 1% of the population per day [23], then 693 trap-days would be required to have a 50% chance of catching a female. The population was taken to be cleared when it was assumed to be undetectable.

#### Costs

An index of the running cost of cattle treatment, covering expendables, depreciation of equipment and monitoring, was made by first recording the cumulative number of square kilometres covered per day. This figure was then multiplied by daily imposed mortality, to allow that high mortalities are more expensive to produce. The procedure is admittedly crude. For example, if a certain number of baits are required to kill 10% of the tsetse per day, somewhat more than double that number are required to kill 20% per day since the additional baits act on an already reduced population. On the other hand, doubling the number of baits hardly doubles the supervision and survey costs. Thus, allowing that these matters roughly compensate for each other, the calculation procedure is judged acceptable for present purposes. The indices of running cost were then divided by 100 to bring them down to convenient levels at which, judging from costings in real terms [17], one unit of the present costs equates very roughly to US$1, and will be considered as such. For artificial baits the running costs were double those for cattle baits [17].

Two distinct phases of operation were costed separately. The initial phase was the period required for the stabilisation of tsetse distribution, *i.e.*, clearing as much territory as possible and bringing the treated area down to the minimum required to form an invasion barrier. The second phase was the maintenance of a barrier, costed as recurrent annual expenditure. Unless stated otherwise, all costs were expressed per kilometre of front. To get the costs per square kilometre cleared or held clear, the costs must be divided by the width of the cleared area.

#### Operational areas

Two main scenarios were modelled and in both cases the area where tsetse control operations was applied (‘operational area’) was adjacent to an invasion source, i.e., an area highly suitable for tsetse where no interventions were applied and hence provides a source of tsetse which can invade the operational area. An imaginary line called the invasion front separated the operational area from the invasion source (Fig. 1).

#### Scenario L – a livestock farming area

Scenario L modelled a settled area consisting mostly of a densely settled area with mixed crop-livestock farming in predominantly good habitat (Fig. 1A). The good habitat continued for 18 km into the operational area; thereafter the habitat became worse, with the mortality of all population components increasing linearly by 25%/km to reach a maximum of treble the standard values at distances >25 km from the front. This simulated the gross change in habitat that often occurs due to the scarcity of hosts, poor vegetation cover and adverse climate, *e.g.*, in moving from a game park through increasingly degraded areas with higher densities of humans and their livestock. Examples of this include transects running: (1) south from the Zambezi valley of Zimbabwe through Makuti (16.3°S, 29.3°E) to the communal lands of Mashonaland, (2) south from the Vwaza Marsh Game Reserve of Malawi through Lake Kazuni (11.1°S, 33.6°E) into settled areas of Rumphi district, (3) north from the Serengeti National Park of Tanzania through Ikoma (2.1°S, 34.6°E) into surrounding farming areas.

#### Scenario W – an isolated wilderness area

In Scenario W, good habitat within the operational area was restricted to a band, 7 km wide at 12–19 km from the invasion front (Fig. 1B). Areas 0–12 km and 19–22 km from the front were poor habitat, with mortality being double the standard. Scenario W is roughly comparable to Mkwaja cattle ranch in Tanzania (5.8°S, 38.7°E), where the invasion source was a game park, from which an imaginary transect went first through mostly grassland, then through a wild wooded area where cattle seldom grazed, before going through grassland again and then onto the very poor habitat of sisal estates [15], [16].

Most consideration was given to Scenario L since its larger expanse of good habitat made it the more difficult for control.

## Results

### Initial populations

**Figure 2:**
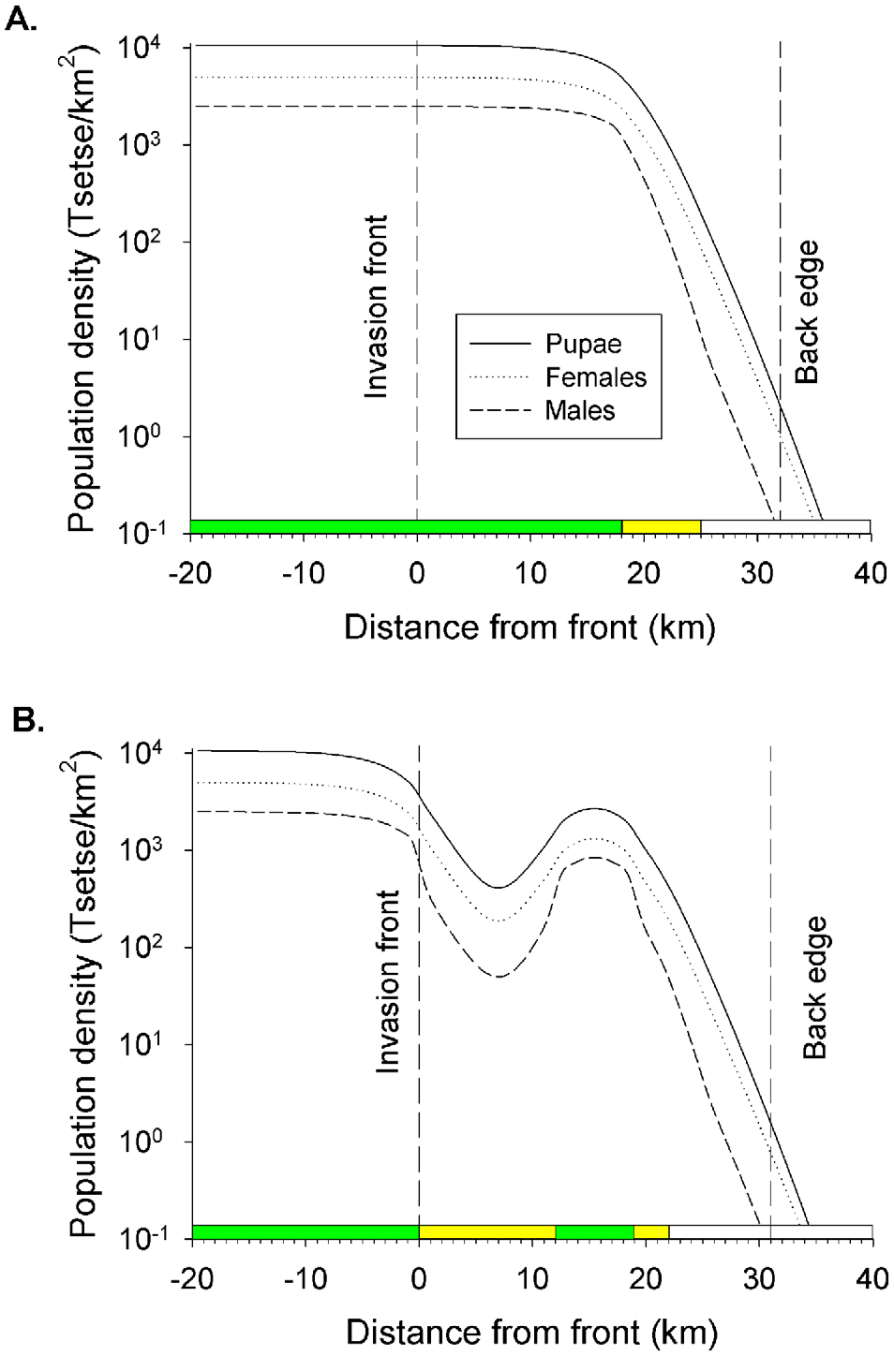
Distribution and abundance of tsetse in the livestock-farming and wilderness scenarios. For both (A) livestock farming and (B) wilderness scenarios, the operational area occurs next to an invasion source, separated by an imaginary line called the invasion front. The other end of the operational area (Back edge) is where the density of males declines to <0.1/km^2^. The bar along the X-axis shows the distribution of good (green sections), poor (yellow sections) and very poor (white sections) habitat. The invasion source is to the left of the invasion front (0 km), and the operational area is between the invasion front and the back edge (32 km).

Prior to any intervention, the number of adult males declined to <0.1 at >32 km from the front in Scenario L (Fig. 2A), and at >31 km in Scenario W (Fig. 2B), so defining the back edges of operational areas 32 km and 31 km wide, respectively. The areas deemed initially infested with tsetse were 3 km wider, at 35 km and 34 km, respectively. Whereas the percent of females in large expanses of good habitat was 67% (*i.e.*, the percent defined for the standard population), the percent was higher in poorer habitats, reaching 92% in the 3 km beyond the back edges, and 79% in the centre of the belt of poor habitat at 0–12 km from the front in Scenario W. This was because many of the flies in the poor habitat were not born there but moved in; females were more likely to enter because they were more mobile per day and also because they lived longer, so being able to move further in their lifetime.

### Even and near-even treatment

#### No gaps

**Figure 3:**
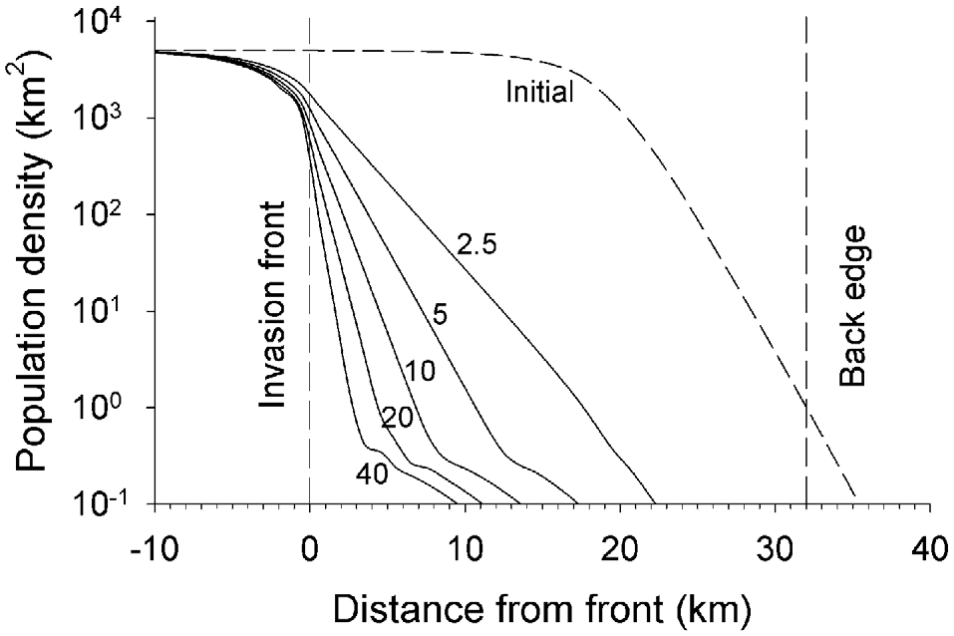
Distribution of female tsetse after 1000 days of control in the livestock farming scenario. Abundance of adult females at various distances from the invasion front of Scenario L (livestock farming area), in the initial population and after 1000 days of control by baits killing 2.5–40% of adults per day. The operational area is where baits were first deployed; the area reduced as the population distribution contracted during the 1000 days.

Simulations of insecticide-treated cattle operated in all bands of the operational area of Scenario L with imposed mortality varying between 2.5 and 40.0% of the tsetse per day for 1000 days showed, not surprisingly, that the width of the cleared area increased as the kill percent increased (Fig. 3). The reduction in population density extended for a few kilometres beyond the area where insecticide-treated cattle were present. This was because the number of tsetse diffusing out of the invasion source was not compensated by an equal number moving back; the number available to move back being reduced by the baits. In that part of the operational area near the front the log-density of tsetse initially declined linearly with increasing distance, but usually tailed off slowly at greater distance because the control measures had been halted there. The upshot was usually the existence of an area where the tsetse population remained just detectable but was not self-sustaining, being maintained by limited immigration from nearer the front.

In the above, and all later simulations, the tsetse abundance far from the invasion source showed a first-order decline with time. For example, with the 10% kill, above, the average modelled densities of females at 15–16 km from the front at 0, 1, 2, 3 and 4 months after treatment were 3626, 757, 99, 20 and 3.4/km^2^, respectively, showing a decline by about 80% per month. As expected, the rate of decline with time was not entirely steady since the age structure of the population took several pupal periods to stabilise, and density dependent reductions in natural deaths came into full play in the second month, *i.e.*, when densities became <10% of standard. After four months, when densities had declined by 99.99%, the daily probability of a female finding a mate had dropped from the original 1.00 to 0.19 and was falling rapidly, to be 0.04 at the end of the fifth month. This would have enhanced greatly the rate of population decline had the population been entirely isolated. However, the rates of decline in the fifth and sixth months were 83% and 82% respectively, *i.e.*, much as before, largely because the population at 15–16 km from the front was being supplemented temporarily by inseminated females invading from the denser, albeit reducing, populations nearer the front.

Baits affected not only the abundance of tsetse populations but also the sex and age structure, as exemplified by the 10% treatment. At 15–16 km from the front, *i.e.*, far from the main invasion source, it was not surprising that the mean age of males and females dropped rapidly after control began, to be 13 and 16 days, respectively, after two months, compared to 20 and 43 days, respectively, at the start. The difference between the mean ages of males and females became less because the mortality imposed by baits far outweighed the differences in the natural mortality of the sexes. Associated with this, the percent of females in the population dropped to 51%, as against 65% initially. At 4–5 km from the front, the mean age of females remained high, being 39 days at five months. This was because the population there was subject to strong invasion; most invaders were older females because they were the more mobile and, in any case, invasion took time so that flies that arrived had to be older than when they left. The upshot was that the female percent in the population there was unusually high, at 77%, despite the presence of baits. Indeed, the effect was more marked the greater the imposed mortality, although then the invasion penetrated less far so it was pertinent to look closer to the front. For example, if the imposed mortality was 40% there were 90% females at 2–3 km from the front after 5 months. In general, an unusually high percent of females is a symptom of significant invasion.

#### Small gaps

**Figure 4:**
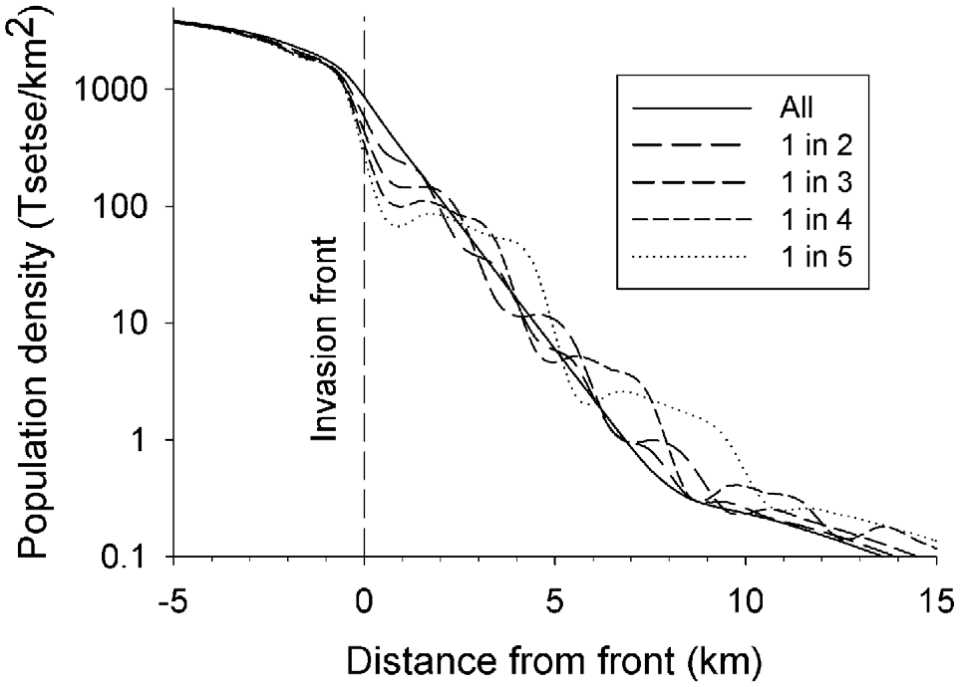
Distribution of tsetse after 1000 days of patchy control in the livestock farming scenario. Abundance of adult females at various distances from the invasion front of Scenario L (livestock farming area), after 1000 days of control by baits killing an average of 10% per day, with treatment being spread over all bands or concentrated into 1 in 2, or up to 1 in 5 bands. The operational area first extended 32 km from the front, but was reduced as the population distribution contracted during the 1000 days; only part of the area is shown.

– The impact of patchy baits was investigated with Scenario L. Simulations were made of treating one band in every two, three, four or five, *i.e.*, leaving untreated areas one, two three or four kilometres wide, respectively, The imposed mortality in the bands was increased to ensure that the mortality averaged over all bands was 10%, *e.g.*, a 50% kill in the treated band when only one band in five was treated. Not surprisingly, the gaps in treatment caused the stabilised density of tsetse to decline irregularly on moving from the invasion front into the operational area (Fig. 4). However, the irregularity was negligible when treating one band in two or three, *i.e.*, when the gaps were 1–2 km wide.

### Timing and costs

#### Scenario L

With the even treatments a rise in the imposed mortality shortened the time to stability, reduced the required width of the invasion barrier and increased the area cleared (Table 1). However, since the higher imposed mortalities involved greater costs per km^2^/day there was relatively little difference in the initial, stabilisation costs. Moreover, although the recurrent costs were higher with the greater imposed mortalities, these were offset by a greater area kept clear. For the uneven treatments at an average of 10% kill, the time to stability and hence the initial and recurrent costs increased with an increase in the unevenness, although there was little or no change in the area cleared, relative to the 10% treatment of all bands. For the special treatment in Table 1, the imposed mortality over the operational area was 10%, except that in the first 4 km from the front the imposed mortality was increased to 40% to form what would eventually become the stand-alone invasion barrier. This combination of treatments reduced the time to stability by only 15 days but increased the stabilisation costs by nearly half, compared to the uniform 10% treatment. Other than ensuring that the invasion barrier was narrower, there was no material benefit from extra kills at the invasion front during the stabilising phase.

**Table 1:** Durations, mortalities and costs of control required to achieve stability at an invasion front.

	Daily mortality (%)				Cost ($)		
Pattern of treated bands	Per treated band	Average for all bands	Days to stability	Treated bands in barrier	For stabilisation	Annual recurrent	Cleared area (km^2^)
All	2.5	2.5	703	20	446	183	13
All	5	5	390	14	463	256	18
All	10	10	203	9	474	329	21
All	20	20	106	6	515	438	24
All	40	40	55	4	554	584	25
1 in 2	20	10	248	5	585	365	21
1 in 3	30	10	296	4	732	438	21
1 in 4	40	10	435	3	1029	438	19
1 in 5	50	10	519	3	1301	548	19
Special: 40% near front, 10% away			188	4	680	584	25

For each row, the days required to stabilise the front, the number of treated bands then in the invasion barrier, and the costs and cleared areas per kilometre of front, are shown, assuming various daily mortalities applied in all bands or in one band in every 2, 3, 4, or 5, in Scenario L. The special treatment is described in the text.

#### Scenario W

The greater expanse of poor habitat in Scenario W meant that the time to stability with each type of bait treatment was lower than in Scenario L, and hence the initial costs were lower. Moreover, the poor habitat near the front was itself a restriction to invasion, so that the required width of the invasion barrier was also lower, thereby reducing the recurrent costs and increasing the area held clear. For example, with an even treatment at 10% kill, stability occurred in 158 days at a cost of $366, compared to figures of 203 days and $474 in Scenario L; the barrier was 7 km wide and the recurrent costs was $256 in Scenario W, compared to figures of 9 km and $329 in Scenario L.

Since tsetse were relatively abundant in and near the central section of good habitat in Scenario W, the control there tended to take longer than elsewhere. For example, with the even 10% treatment, above, the 158 days to stabilisation were set by the time needed to deal with the tsetse in the good habitat, whereas the population in the poor habitat stabilised in 136 days. Moreover, even the relatively quick stability in the poor habitat took longer than it would have if the population there had not for some while been invaded from the more persistent population in the good habitat. Hence, it made sense to increase the kill rate in and near the good habitat, to speed and synchronise the operations. For example, if the imposed mortality was raised to 20% in the good habitat and also in the one band outside each of its edges, and was left at 10% elsewhere, then the population in the good habitat was cleared in 92 days, and overall stability occurred at 105 days. The whole clearance and stabilisation operation was 51 days quicker than with the 10% treatment throughout, and the cost was $28 less. Against this, the recurrent costs of barrier maintenance were incurred earlier, at $36 for the 51 days, so that costs were $8 greater overall. This illustrates the general principle that provided no great gaps occurred in bait cover, then variation in kill percents changed the timing more than the costs.

#### Other scenarios

In all of the above simulations the habitat immediately inside the invasion source was good, so supporting there a dense population associated with strong invasion pressure which required a broad invasion barrier. However, in some circumstances, such as the heavily settled parts of northern Zimbabwe, the invasion pressure is low because poor habitat in the operational area extends far into the invasion source. Hence, in a number of separate simulations the operational area consisted of poor habitat which extended either 0 km, 5 km, 10 km or 15 km into the invasion source, so that the initial female populations against the front consisted of 2121, 260, 32 and 4/km^2^, respectively. Allowing that the imposed mortality in the barrier was 10%, the required width of the barrier was 7 km, 6 km, 4 km, and 2 km respectively. The invasion pressure had to be reduced by about 99% before the width of the invasion barrier was halved.

### Consequences of large central gaps

**Figure 5:**
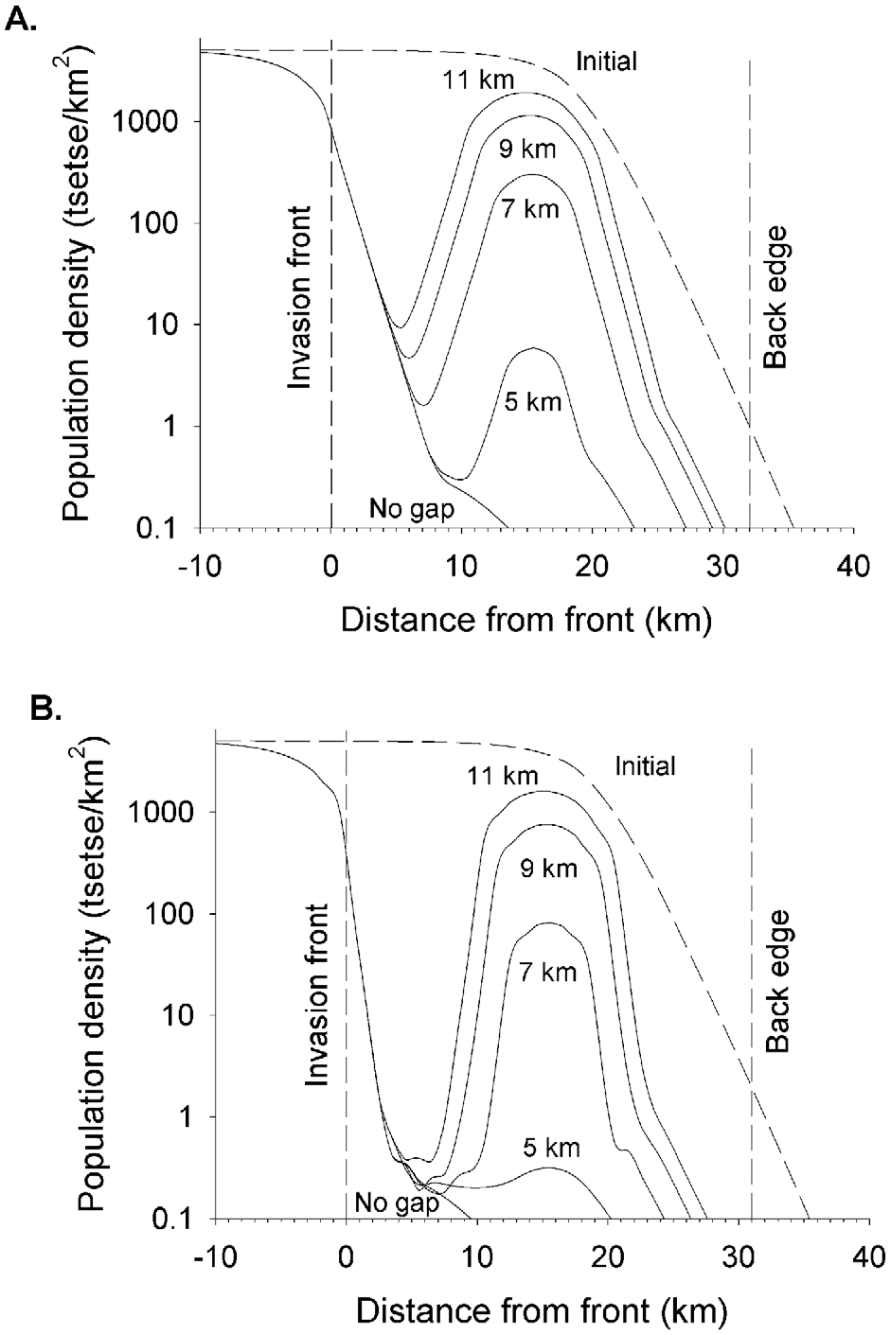
Impact of patchy tsetse control with different imposed mortalities in the livestock farming scenario. Abundance of adult females at various distances from the invasion front of Scenario L (livestock farming area), in the initial population or after 1000 days of control by baits deployed with no gap or gaps 5–11 km wide centred at 15–16 km from the front, for daily mortalities of 10% (A) or 40% (B) in the treated areas.

In Scenario L it was taken that insecticide-treated cattle were not present in areas 5–11 km wide, centred on the band at 15–16 km from the front. After 1000 days with a 10% kill in treated areas (Fig. 5, A) a residual population remained in the untreated areas, and, not surprisingly, the population there was greater the wider the gap. Increasing the imposed mortality to 40% in the treated areas of Scenario L roughly halved the residual population in the gap and caused the population to decline more sharply on moving away from the gap (Fig. 5, B). If operations were continued beyond the 1000 days the residual populations in the gaps 5–7 km wide did eventually disappear. For example, with a 10% kill outside the gaps the residual population in the 5 km-wide gap expired after 1516 days, and after 4182 days with the 7 km-wide gap. When the imposed mortality outside the gaps was raised to 40% the times required decreased, being 1081 days and 2660 days, respectively.

### Treating large central gaps

If the gaps in the distribution in insecticide-treated cattle are at least 5 km wide, then merely increasing the imposed mortality produced by the cattle – even to a level (40%/day) approaching the maximum possible (50%/day) - outside of the gaps is a slow or ineffective means of dealing with the flies inside (*cf* A and B in Fig. 5). A better solution is to use targets to fill the gaps. If the imposed mortality due to the targets is kept the same as that of the cattle, the timing of the control and the width of the invasion barriers are exactly as if the cattle treatment were used throughout. Only the cost varies, since the unit cost of targets is double that of cattle. For example, if targets substitute for cattle in a quarter of the whole treated area for the whole operational period, then the overall costs increase by half. However, deploying and servicing targets is relatively inconvenient, so in practice the density of targets might be less than cattle and/or the timing of target deployment and cattle treatment might not be perfectly synchronised. Modelling these possibilities showed, not surprisingly, that the earlier the targets were started the sooner stability was achieved after the inception of cattle treatment, and the lower were the total costs to stabilisation (Table 2). However, provided the targets were not deployed late, the total costs were less than double those of using a uniform 10% kill by cattle throughout (Table 1).

**Table 2:** Cost-effectiveness of using targets and cattle to control tsetse.

Start of targets relative to cattle	Daily mortality at targets (%)	Days to stability	Costs for stabilisation ($)		
			Cattle	Targets	Total
180 days ahead	2.5	494	719	116	835
	5.0	259	388	152	540
0 days ahead	2.5	559	845	96	941
	5.0	314	504	108	612
180 days later	2.5	696	1092	89	1181
	5.0	469	781	99	880

For each row, the days required to stabilise the tsetse distribution in Scenario L, and the costs of stabilisation are shown. The simulations assumed that insecticide-treated cattle, with a daily mortality of 10%, were deployed in all parts of the operational area, except for a central section 7 km wide where targets imposing various mortalities were started at various times in relation to the start of cattle treatments. Days to stability are counted from the start of cattle treatments.

### Gap position

In the above simulations the large gaps in cattle treatments were in the middle of the operational area. Failing to fill a gap was less serious when the gap was near the back edge, *i.e.*, where the population was struggling to persist, instead of within a few kilometres of the invasion front. Indeed, it was possible to achieve successful eradication without treating a large part of the rear of the operational area. For example, in Situation L with 10% imposed mortality, 10 km of the rear could be left untreated without increasing the time to stabilisation. This also reduced the cost by $58 – not much since even if cattle were operated in the rear they were not required for long. Times and costs rose if more of the rear were left, *e.g.*, with 15 km untreated the time increased by 485 days and costs by $537.

### Increased realism

Although the above simulations have exposed the basic theory of bait control they are unrealistic in several ways. First, the modelling assessed tsetse abundance instantly and precisely, so that modelled control could be stopped exactly when and where it became unnecessary, but in practice the surveys of abundance take several months, during which caution demands assuming the worst and maintaining control. Second, in the field it may be suspected that the tsetse infestation is expanding, in which case it is safest to have little or no back gap. Third, while it was assumed that the control could be applied instantly everywhere, in truth the shortage of materials and supervisory capacity may require progressive implementation, in a “rolling carpet” strategy from the back edge to the invasion source. Fourth, it may be impractical to stop control on a band-by-band basis; blocks of bands 5–25 km wide are more likely to be considered. Finally, it is unwise to produce an invasion barrier of the bare minimum width; a few extra kilometres insures against temporary breakdowns in barrier upkeep. It also means that tsetse disappear from the ‘invasion zone’ – an area where the tsetse present comprise invading flies only - thereby increasing the cleared area and forming a zone where surveys would give a clearer warning of barrier breakdown. No tsetse should then be caught in the invasion zone and thus it is not required to make the difficult distinction between catching a few in one month, and one or two more than a few in the next.

To simulate greater realism it was taken that in any given block of bands the control extended for three 30-day months after the first complete month in which the maximum density in the block dropped to <0.1 males/km^2^, except that if the maximum density in the block was below the critical level on the first day of the control period in that block then the control there lasted four months. Two distinctive plans were then considered for Scenario L, involving insecticide-treated cattle (Table 3). In Plan F, aimed at fast control, the imposed mortality in treated areas was 40%, and a back gap 3 km wide was allowed, with the whole of the operational area being treated at once. Plan S was slower and more cautious, employing no back gap and an imposed mortality of 10% applied in a rolling carpet that started in a relatively small block, assuming that the control personnel wanted to prove the techniques before increasing the block size. Both plans employed a final phase which maintained an invasion barrier that was 30–50% greater than the minimum, for 12 full months. This was to allow full stock to be taken by surveys, perhaps before rolling on into the invasion source or handing the barrier upkeep to local operatives.

**Table 3:** Duration, cost and impact of fast (F) or slow (S) control operations.

Plan	Phase	Km from front	Months duration	km^2^ cleared	Cost ($)
F	1	0–29	5	29	1740
	2	0–6	12	0	864
	Total		17	29	2604
S	1	20–35	5	7	225
	2	10–30	4	3	240
	3	0–25	8	14	600
	4	0–12	12	0	432
	Total		29	24	1497

The distance of operations from the invasion front of Scenario L, their duration and the costs and area cleared per kilometre of front, in various phases of Plans F and S detailed in the text.

Not surprisingly, the costs were greater than the stabilisation cost in previous modelling, since substantial safety precautions and the costs of maintaining the barrier for a year were included. Expressed per square kilometre cleared and held clear, the cost for Plan F was $90, compared to $62 for Plan S. The greater expense of Plan F might easily be justified by the sooner benefits associated with quicker clearance; the greater cleared area could be important if the benefits per square kilometre were high. Judging from the costs of control during the clearance phases of Plan S, the cost of progressive and cautious clearance of a further 10–15 km per year, with an advancing barrier, are about double those of maintaining a static barrier for a year.

### Vigilance

The population at and near the back edge of the invasion barrier must be monitored to give early warning of any breakdown in control, and so allow correction before the population spreads far. For example, with the 13 km-wide barrier involving the 10% imposed mortality, above, let it be taken that the imposed mortality drops to 2%, perhaps because the insecticide becomes less effective due to application errors. Then the population at 1, 2, 4, 8 and 12 km outside the back edge of the barrier becomes self-sustaining after 91, 106, 143 248 and 463 days respectively. After a year, virtually all of the territory that was the most difficult to clear would be lost, although the extensive surveys required to detect this failure might take a further six months in which more expansion would occur. By then it would be necessary to repeat almost all of the previous control.

If continuous surveys are conducted within the barrier there will be a month or so of warning that the control is awry, allowing the situation to be rectified without increasing the treated area. If the population is allowed to extend for 1 km (after 91 days) behind the barrier, then the matter could be put right by returning the imposed mortality to 10% within the barrier and applying it in the 1 km where the self-sustaining population has spread, together with a further 3 km for safety. The situation is then restored after 46 days, so that the barrier can be returned to the normal width after five months, allowing three full months of added control while surveys confirm that corrective measures have indeed been effective. The cost of operations outside the barrier is $75, with little increase in the threat of disease. However, if the treatments are put right only after the population has extended for 8 km (after 248 days), the correction period is 94 days, making a total of seven months of control to cover also the surveys. The cost is $231, and the disease risk has been longer and more widespread. Present indications for the speed at which tsetse invade after reducing the restrictions, and the ease with which prompt action can restore the situation, accord with field experience [24].

## Discussion

The modelling performed here uses inputs for the dynamics of births and deaths that are essentially the same for all tsetse [18]. However, the inputs for the daily displacement refer primarily to savannah species, such as *G. morsitans*. For these species, the present results suggest that in the typical farming areas of East and Southern Africa, where cattle are abundant but unevenly spread, small (<3 km) cattle-free pockets within a larger operational area are unlikely to have a deleterious impact on the efficacy of using insecticide-treated cattle to control tsetse. Larger pockets will delay and/or prevent the achievement of effective control even when there are high densities of cattle adjacent to the pockets. In these circumstances, the simplest and most cost-effective strategy is to deploy insecticide-treated targets where cattle are absent.

The usefulness of the model and its outputs is to be judged by the extent to which the general pattern of its outputs accord with field observations, as below.

### Density

In areas far from an invasion source, tsetse abundance showed a first-order decline with time, as observed in the field with a variety of tsetse species [11], [14], [25], [26]. Similarly, in areas subject to constant reinvasion tsetse can penetrate various distances into an operational area according to (i) the density of tsetse in the invasion source and (ii) the distribution and abundance of baits. In Zimbabwe, tsetse were detected up to 5 km into an operational area where baits exerted a daily mortality of ∼10% in accordance with the present simulations (see Fig. 3). As a consequence of invasion, small areas (i.e., <10 km across) cannot be cleared of Morsitans-group tsetse using standard densities of baits (e.g., 4 insecticide-treated targets or cattle/km^2^) as shown by the present simulations (Fig. 5A) and seen in practice [11], [27], [28].

### Sex composition

The simulations showed that changes in mortality due to natural factors (e.g. habitat) or control efforts will alter the sex composition of tsetse populations. These results accords with field observations of high percentages of females in catches in poor habitats, *e.g.*, [29], although before the demonstration that females move more than males [30], [31] it was usually considered that high proportions of females indicated starving populations [32]. Since it was mostly females that diffused from the good to poor habitats, the model's output for the proportion of females in good habitat was slightly lower than the standard 67% if there was poorer habitat within a few kilometres.

### Age structure

Baits also affected age structure. For instance, for tsetse 15–16 km from the invasion front, *i.e.*, far from the main invasion source, with the standard bait density (10% daily mortality rate) applied under Scenario L, the mean age of males and females dropped to 13 and 16 days, respectively, after two months, compared to 20 and 43 days, respectively, at the start. This has important epidemiological implications particularly for the transmission of *Trypanosoma brucei* spp, the causative agents of sleeping sickness, which requires a development period of ∼20 days in the fly. Again, these results accord with field observations where the mean age of females caught from traps declined following the start of control operations [33].

The general agreement between the outputs of Tsetse Muse and reliable field data available for populations of medium to high density offers *prima facie* evidence that the modelling is not seriously awry with very sparse populations for which satisfactory field data are not available. The worst unknowns are the density-dependant changes in population dynamics [18]. However the high mortality imposed by baits are sufficient to swamp any density-dependant reductions in natural mortality, so elimination is achievable even if there were no natural deaths at low population density.

### Lessons learned

The results provide several new insights that have important implications for the control of tsetse and trypanosomiasis.

First, since population decline due to baits is logarithmic, the rate of decline seems to decrease on a normal scale. Such observations can be misinterpreted as being indications that an operation is becoming less effective as control proceeds. Related to this general phenomenon, because baits reduce tsetse populations rapidly on a normal scale, there is the danger of believing mistakenly that control efforts can be relaxed to complete the task.

Second, while the present estimates of control costs are crude, they do highlight the general pattern of how costs change with technical options. The main finding is that without gross variation in costs there is much latitude in tailoring robust bait measures to suit the required rate of control, implementation capacities, risks and economic conditions, in a range of operational areas.

Third, heterogeneities in the deployment of baits are inevitable with insecticide-treated cattle - the behaviour of cattle along with the demands of providing adequate grazing, water and security means that cattle are never evenly distributed. The present results provide a preliminary framework for understanding the likely implications of the problem and possible solutions. In particular, patchy distributions of baits will be serious if the gaps occur in good habitat, are broader than about 10 times the daily displacement of tsetse (e.g. ∼3 km for *G. morsitans*, and are not recognised until many months after control begins. The problem can be solved by deploying targets in the gaps since their mode of operation is closely similar to cattle baits – both offer continuous control of resident and invading flies for as long as necessary, and can be planned to work at roughly similar rates. By contrast, treating the gaps by sequential spraying of non-residual insecticide offers no protection against invasion since during and after each application the flies can enter from unsprayed areas nearby [31]. This ensures that two months later, when all applications are complete, a residual population remains. The problem could be overcome by extending the spraying into much of the surrounding area, to kill potential invaders, but this would be very expensive [17], especially if the gap is relatively small and so requires the high fixed costs of an aerial spraying cycle to be spread over a small area.

Finally, the indication that progressive clearance by baits is not grossly more expensive than mere barrier up-keep reinforces the prospect of clearing tsetse from the whole of international fly-belts, thus eventually avoiding the invasion problem [6]. The speed of clearance could be enhanced by a combination of baits and large aerial spraying campaigns [4] with the spraying occurring mostly in places where cattle are absent and ground access is difficult.

## Supporting Information

Table S1 Features of the tsetse population in good habitat before any interventions.
(DOC)
