# Supplementary material for: Is the Even Distribution of Insecticide-Treated Cattle Essential for Tsetse Control? Modelling the Impact of Baits in Heterogeneous Environments
Source: PLoS Negl Trop Dis. 2011 Oct 18;5(10):e1360. doi: 10.1371/journal.pntd.0001360 (PMC3196476; doi:10.1371/journal.pntd.0001360)
Supplement: Table S1 — Features of the tsetse population in good habitat before any interventions. (DOC) [file pntd.0001360.s001.doc]

Table S1. Features of the tsetse population in good habitat before any intervention.

|  | Males | Females | Source ^e^ |
| --- | --- | --- | --- |
| Adults per km^2^, all ages | 2500 | 5000 | [34] |
| Mean adult age | 24 | 44 | M [18] |
| Pupal period | 28 | 26 | [18] |
| Age at sexual maturity ^a^ | 5 | 3 | [34]* |
| Daily probability that a virgin female will meet 1 male/km^2^ | - | 0.1 | M [20] |
| Age at first larval production, if mated ^a^ | - | 16 | [18] |
| Interlarval period | - | 9 | [18] |
| Maximum adult life span | 89 | 178 | [34] |
| Natural mortality (%) Pupae, per pupal duration | 25 | 25 | [18] |
| Eggs/larvae ^c^ | 5 | 0.5 | [18] |
| Adults per day Average ^c^ | 6.14 | 3.07 | M [18] |
| First day | 14.21 | 13.62 | M [18] |
| Young adult | 2.84 | 1.36 | M [18] |
| Last full day | 8.52 | 4.09 | M [18] |
| Mean adult displacement, m/day Average ^d^ | 249 | 367 | [21] [31] |
| First day | 100 | 200 | E [21] |
| Young adult | 300 | 600 | E [21] |
| Last full day | 200 | 400 | E [21] |

Average daily temperature is assumed to be 25^o^C; all ages and periods are in days.

^a^ Females that mated less than five days, i.e., about half an interlarval period, before the age at which larval production was normally due did not produce a larva until the next due age.

^b^ If an egg or larva died before completing its development its mother did not start to develop a new egg until her next due age.

^c^ The death rates for the first day of adult life increased linearly to become the young adult rate at age 10-15 days for males and 10-50 days for females. Thereafter, the rates increased linearly to those for the last full day.

^d^ Flies were modelled as occurring in the centre of each 1km section of the transect. A proportion of them displaced between adjacent centres each day, to give the required rate of daily displacement. The rate on the first day increased linearly to become the young adult rate at age 10-25 days for males and 10-50 days for females. Thereafter, the rates declined linearly to those for the last full day.

^e^ M: exact value calculated from by the model itself but within the range expected from published data. E: estimated parameter that makes little material difference to the patterns of the outputs.

* [34]. Glasgow, JP (1963) The distribution and abundance of tsetse. Oxford: Pergamon, 241 p. For all other references, see main text.
